# Microenvironmental pH and Exosome Levels Interplay in Human Cancer Cell Lines of Different Histotypes

**DOI:** 10.3390/cancers10100370

**Published:** 2018-10-05

**Authors:** Mariantonia Logozzi, Davide Mizzoni, Daniela F. Angelini, Rossella Di Raimo, Mario Falchi, Luca Battistini, Stefano Fais

**Affiliations:** 1 Department of Oncology and Molecular Medicine, Istituto Superiore di Sanità, Viale Regina Elena 299, 00161 Rome, Italy; mariantonia.logozzi@iss.it (M.L.); davide.mizzoni@iss.it (D.M.); rossella.diraimo@iss.it (R.D.R.); 2 Neuroimmunology Unit, IRCCS Santa Lucia Foundation, 00179 Rome, Italy; df.angelini@hsantalucia.it (D.F.A.); l.battistini@hsantalucia.it (L.B.); 3 National AIDS Center, Istituto Superiore di Sanità, Viale Regina Elena 299, 00161, Rome, Italy; mario.falchi@iss.it

**Keywords:** acidity, exosomes, microenvironment, nanoparticle tracking analysis, biomarkers

## Abstract

Exosomes are extracellular nanovesicles primarily involved in the pathogenesis of many diseases including cancer. This study was set out from recent evidence that extracellular acidity may increase the exosome release by cancer cells. However, this preliminary evidence did not provide solid information on whether the pH-dependent exosome over-release represents a common feature of all cancers. To the purpose of demonstrating that cancer acidity is a major determinant in inducing an increased exosome release by human cancer cells, we evaluated human tumor cell lines deriving from either colon, breast, prostate cancers, melanoma, or osteosarcoma. All cell lines were cultured in either the current 7.4 pH or the typical pH of cancer that is 6.5. The levels of released extracellular vesicles were measured by protein counts, nanoparticle tracking analysis (NTA), and nanoscale flow cytometry. The results showed that pH 6.5 induced a remarkable increase in exosome release, and buffering the medium significantly reduced the exosome release in all cancers. With these results, we provide, for the first time, evidence that tumor acidity and exosome levels represent common cancer phenotypes.

## 1. Introduction

Exosomes are natural nanoparticles (40–180 nm), bound by a lipid membrane, which are released by all cells of our body both under normal and pathological conditions, and are responsible for intercellular communication, as well as for the elimination of toxic substances and drug delivery [1,2]. Exosomes belong to a wider class of extracellular vesicles (EVs) that also includes microvesicles (from 180 nm to 1 µm) and apoptotic bodies (from 1–5 µm), from which they differ in biophysical, biochemical, and molecular composition [3,4,5].

The exosome membrane has a characteristic lipid composition enriched in cholesterol and sphingomyelin, two lipids typically abundant in lipid rafts [6], in ceramide, important for the insertion of microRNAs into exosomes [7], and in Bis(monoacylglycero)phosphate (BMP), an exosome-specific marker [8].

Exosomes also shuttle various molecules into target cells, transmitting their load of specific molecules (lipids, proteins, DNA, messenger RNA (mRNA), or microRNA) capable of modulating physiological or pathological processes, including tumor progression [9,10,11]. Together with modulating a range of normal functions, exosomes may be considered as a way our body exploits to interconnect organs and compartments [4]. However, these natural nanovesicles are actively involved in tumor progression, and inhibition of tumor exosome release may represent an antitumor strategy [1,12,13]. The tumor microenvironment is typically acidic, and extracellular acidity is a common phenotype of almost all tumors [14]. Actually, the acidic extracellular microenvironment represents per se a defined characteristic of malignancy, which cannot be considered separately from the intrinsic molecular characteristics of cancer cells. Tumor pH may range from 6.0 to 6.8, and the level of acidity is directly related to the tumor degree of malignancy [14,15,16,17,18,19,20]. Among the dominant phenotypes of tumors, which are actually common to all human cancers, are hypoxia, acidity, and low nutrient supply. A dominant aspect of the tumor microenvironment is the so-called "Warburg effect", characterized by the use of sugar fermentation independently of oxygen presence [21,22,23,24]. This phenomenon leads to the conversion of a glucose molecule into two molecules of lactic acid and 2H^+^ to produce 2 ATP, compared to the 36 ATP produced by oxidative metabolism [22,23], thus inducing the accumulation of lactic acid and H^+^, and therefore, the reduction of extracellular pH [25]. Furthermore, high levels of carbon dioxide produced during mitochondrial respiration of oxygenated tumor cells may also contribute to a substantial release of H^+^ into the tumor environment [25]. Since exosomes are present in all body fluids (blood, saliva, cerebrospinal fluid, breast milk, and urine) [4,26], searching for exosome-associated biomarkers in these fluids is potentially a more efficient, less invasive, and less expensive system than traditional diagnostic methods [26,27,28,29]. Extracellular acidity induces a selective pressure leading to selection of cancer cells that are able to survive in such a hostile microenvironment. Typical phenomena related to this selection are the increase in exosome release [28] and the resistance to a wide variety of drugs, including all drugs commonly used in the treatment of tumors [1]. All in all, cancer cells of any histotype are equipped to survive in an acidic microenvironment, a condition that does not allow the survival of normal cells [17]. Among the mechanisms involved in this ability to resist in a hostile environment are proton pumps, which avoid cytosolic acidification and enhance the acidification of the extracellular environment [13,30]. In fact, to survive and proliferate in acidic conditions, tumor cells upregulate proton exchangers and transporters (mainly vacuolar H^+^ ATPase (V-ATPase), Na^+^/H^+^ exchanger (NHE), monocarboxylic transporters (MCT), and carbonic anhydrases (CA), which actively extrude protons in excess, thus avoiding the intracellular accumulation of toxic molecules [25,30].

Some features of the tumor microenvironment may be key factors in the paracrine regulation of exosome traffic within the tumor mass. In fact, a low-pH condition is a hallmark of tumor neoplasia that potentially influences exosome release, thus promoting and maintaining the spread and progression of cancer. In this sense, it represents a system of malignancy paracrine diffusion [6]. Preliminary data showed that melanoma cells cultured at pH 6.7 release a significantly greater number of exosomes, when compared to the same cells cultured at pH 7.4 (physiological pH) [6,28], simulating what conceivably occurs in tumor patients [28].

Recent technologies provided the opportunity to have a precise measurement of exosome level using the nanoparticle tracking analysis (NTA). The purpose of this work was, therefore, to use NTA and other complementary approaches to quantify exosome release under different pH conditions. The aim was to provide an additional diagnostic tool able to evaluate the number of plasma (thus, circulating) exosomes in patients with tumors of various histotypes, allowing the identification of what was recently defined as liquid tumor mass [13,28,31]. In this study, we showed an increase in exosome release at acidic pH (6.5) as compared to physiological conditions (pH 7.4), independently of the tumor histotype (metastatic prostate carcinoma LNCaP, metastatic melanoma Me30966, SaOS2 osteosarcoma, SKBR3 metastatic breast adenocarcinoma, and HCT116 colorectal carcinoma). As mentioned, we used nanoparticle tracking analysis (NTA) together with nanoscale flow cytometry [28,32,33], thus providing clear experimental evidence on the crucial role of tumor pH in conditioning exosome production, and suggesting that anti-acidic or alkalizing treatments may represent not only a valid anti-tumor therapeutic approach but also a highly effective tool for considerably reducing the metastatic diffusion of tumors.

## 2. Results

### 2.1. Protein Quantification and Characterization by Western Blot Analysis for Housekeeping Markers of Exosomes

**Figure 1:**
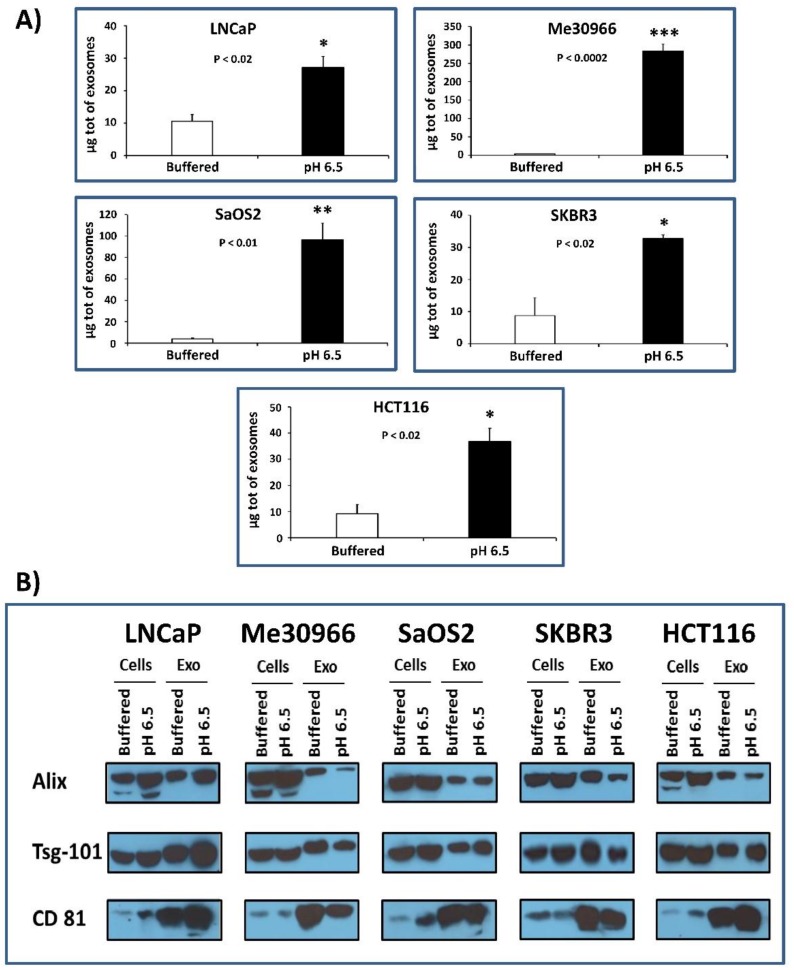
Protein quantification and characterization by Western blot analysis for housekeeping markers of exosomes. (**A**) Protein analysis of exosomes purified form several tumor cell lines (LNCaP, Me30966, SaOS2, SKBR3, and HCT116) cultured in both buffered and pH 6.5 conditions. Protein quantification was performed with the Bradford protein assay, and the exosomes were lysed using 3-((3-cholamidopropyl) dimethylammonio)-1-propanesulfonate (CHAPS) buffer. Means ± standard error (SE) of three different experiments are shown. The *p*-values were <0.02 in all cellular lines cultured in pH 6.5 with respect to buffered conditions. (**B**) Western blot analyses of alpha-1,3-mannosyltransferase (ALG-2)-interacting protein X (Alix), tumor susceptibility gene 101 (Tsg101), and cluster of differentiation 81 (CD81) proteins, performed in total protein extracts of several tumor cell lines (LNCaP, Me30966, SaOS2, SKBR3, and HCT116), cultured in buffered and pH 6.5 conditions, and exosomes purified from supernatants of the same cells. * *p* < 0.02, ** *p* < 0.01, *** *p* < 0.0002.

**Figure 2:**
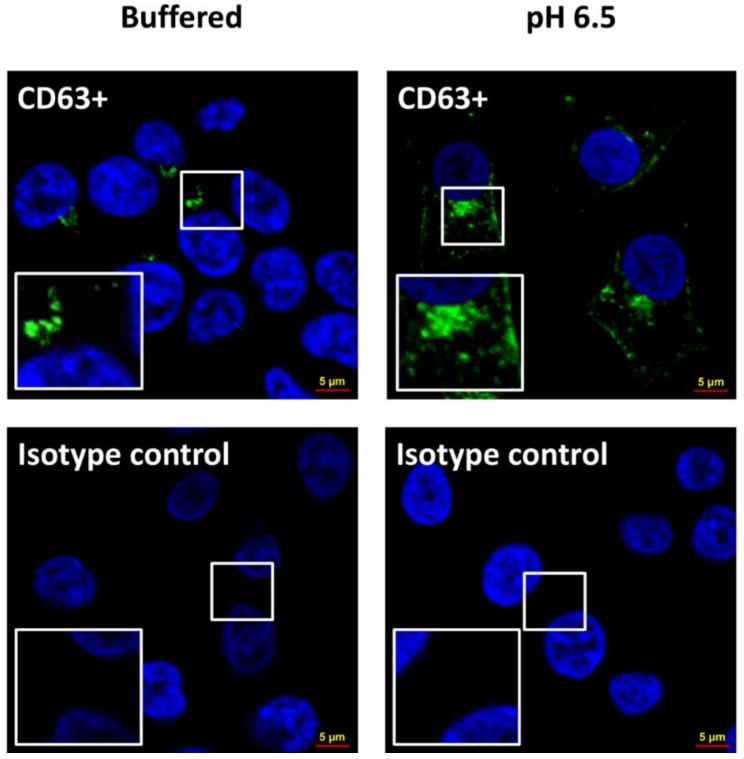
Confocal microscopy of tumor cells cultured in buffered and pH 6.5 conditions. The cells, after being fixed in 3% paraformaledehyde, were labeled with CD63 monoclonal antibody (MEM-259), and Alexa Fluor 488 with a concentration of 1:25 for 2 h at room temperature; then, they were labeled with diamidino-2-phenylindole (DAPI) and observed with a confocal microscope. (**Left**) In upper panel, tumor cells cultured in buffered conditions labeled with CD63; in lower panel, the same cells with isotype control. (**Right**) In upper panel, tumor cells cultured in pH 6.5 conditions labeled with CD63; in lower panel, the same cells with isotype control.

Several tumor cell lines (LNCaP, Me30966, SaOS2, SKBR3, and HCT116) were grown in buffered medium (pH 7.4) and simultaneously stabilized and cultured at pH 6.5 conditions. The cell mortality test (Trypan blue assay) conducted by cytofluorimeter analysis showed that all cell lines in the two different culture conditions had no differences in mortality, which was actually generally low (1–2%; data not shown) [16]. Then, after five days of culture, exosomes were isolated from the supernatants of all five tumor lines cultured at pH 7.4 and pH 6.5 through serial ultracentrifugation in order to analyze protein quantification. Protein analysis was performed with the Bradford protein assay and the exosomes were lysed using 3-((3-cholamidopropyl) dimethylammonio)-1-propanesulfonate (CHAPS) buffer. The results were obtained by normalizing with the number of live cells contained in the culture from which the exosomes were purified; the statistical analysis was performed using the unpaired *t*-test with the GraphPad Prism 6 program. Figure 1A shows that cells grown at acidic pH produce a greater amount of exosomes as compared to the same cells grown in buffered medium. In particular, in SaOS2 and in Me30966 cells, the exosome release was 24–78-fold more in acidic conditions as compared to 7.4 pH conditions, while it was fourfold more in both SKBR3 and HCT116 cells, and threefold more in LNCaP cells. We characterized the exosomes released in the culture media to verify the presence of typical exosome markers (such as alpha-1,3-mannosyltransferase (ALG-2)-interacting protein X (Alix), tumor susceptibility gene 101 (Tsg101), and cluster of differentiation 81 (CD81)) using Western blot analysis (Figure 1B); in fact, an equal amount of exosomes purified from the examined cell lines showed comparable amounts of typical markers. To follow up the cellular changes during the pH variations, we used confocal microscope analysis. The results showed that the acidic microenvironment induced modifications in morphology and protein expression in all tumor cells. Figure 2 clearly shows an example of the cell viability after a period of 3–4 weeks at pH 6.5; all cells were perfectly viable, but with a clear higher intensity of CD63 expression in tumor cells cultured at pH 6.5 with a massive cytoplasmic localization as compared to the same cellular histotypes grown at pH 7.4, suggesting an increase in the activity of the endosomal compartment, in turn, leading to exosome formation and extracellular release.

### 2.2. Nanoscale Flow Cytometry Quantification and Characterization of Exosomal Preparations Obtained from Supernatants of Tumor Cells Grown under Different pH Conditions

The exosomes isolated from the supernatants of tumor cells cultured in two different conditions (buffered and pH 6.5 medium) were analyzed using nanoscale flow cytometry (Cytoflex) for the presence of typical exosomal CD81 and CD9 markers, respectively labeled in allophycocyanin (APC) and in phycoerythrin (PE). PE has maximum excitation at 496 nm and maximum emission at 576 nm; thus, it absorbs slightly blue-green and yellowish light and emits yellow-orange light. APC absorbs and emits red light (650 and 660 nm max, respectively). Nanoscale flow cytometry is a very promising approach for the characterization of nanovesicles, as demonstrated in a recent study [28]. 

**Figure 3:**
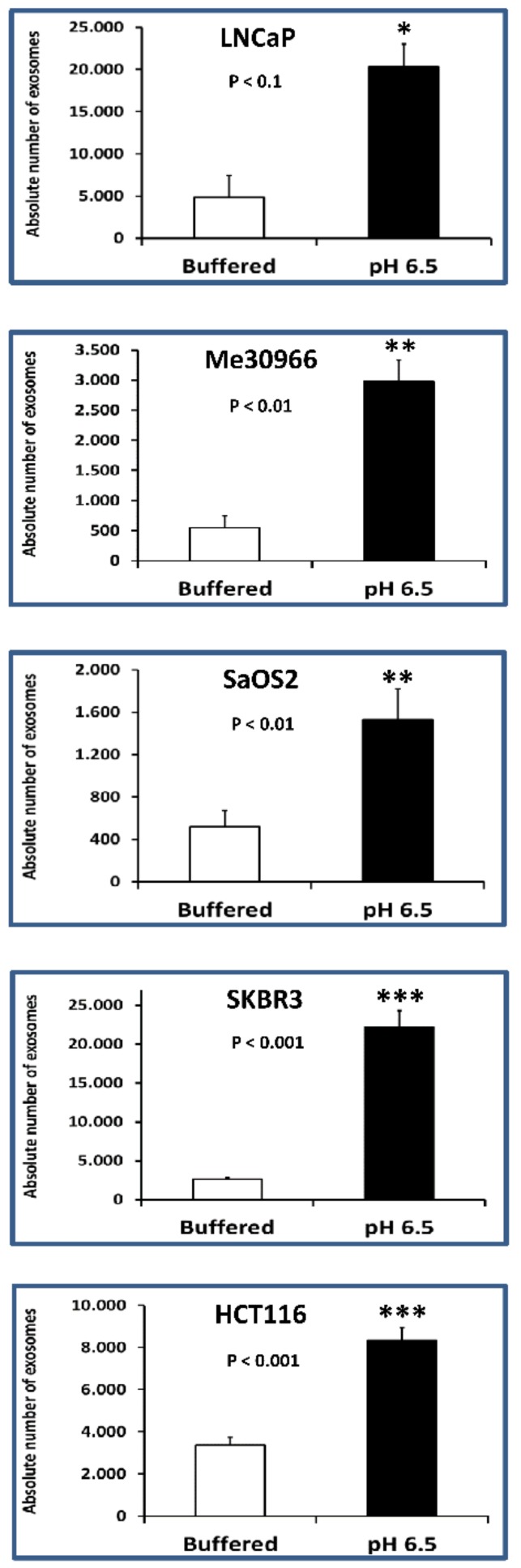
Nanoscale flow cytometry of exosomes in tumor cells cultured in buffered and pH 6.5 conditions. The cytometer was calibrated using a mixture of non-fluorescent silica beads and fluorescent (green) latex beads with sizes from 110 nm to 1300 nm. The exosome preparation derived from several tumor cell line (LNCaP, Me30966, SaOS2, SKBR3, and HCT116) supernatants cultured in different pH cell culture conditions (buffered and pH 6.5 medium) were stained with anti-CD9 and anti-CD81 antibodies and analyzed using flow cytometry. The double-positive events were then analyzed for their size, based on the calibration with beads. Cumulative data are shown of the absolute number of CD9^+^/CD81^+^ exosomes of size less than 180 nm recovered from the samples at pH 6.5 as a function of those recovered from samples at pH 7.4. Data are expressed as means ± SE of three independent experiments. The *p*-values were <0.1 in all cellular lines cultured in pH 6.5 with respect to buffered conditions. * *p* < 0.1, ** *p* < 0.01, *** *p* < 0.001.

Double-positive events were counted and analyzed by size. The results showed that the number of double-positive exosomes smaller than 180 nm was greater when the cells were cultured at pH 6.5. Figure 3 shows the absolute average number of exosomes (sizes less than 180 nm) obtained from either 7.4 or pH 6.5 conditions. 

In particular, in HCT116, SaOS2, LNCaP, Me30966, and SKBR3 cells, the exosome release was 3–8-fold more in acidic conditions as compared to pH 7.4 conditions (Table 1). The statistical analysis was performed using the unpaired *t*-test with the GraphPad Prism 6 software.

The results obtained using nanoscale flow cytometry suggest that cells grown in acidic pH (6.5) release, on average, 4.6-fold more exosomes than the same cells grown in physiological (buffered) medium. These data are the result of the ratio between the average of the absolute number of CD9^+^/CD81^+^ exosomes released by all tumor histotypes cultured at pH 6.5 and the averages of the absolute number of CD9^+^/CD81^+^ exosomes produced by the same cells grown in buffered medium. The results are summarized in Table 1.

### 2.3. NTA Quantification of Exosome Preparations Obtained from Supernatants of Tumor Cells Grown under Different pH Conditions

**Figure 4:**
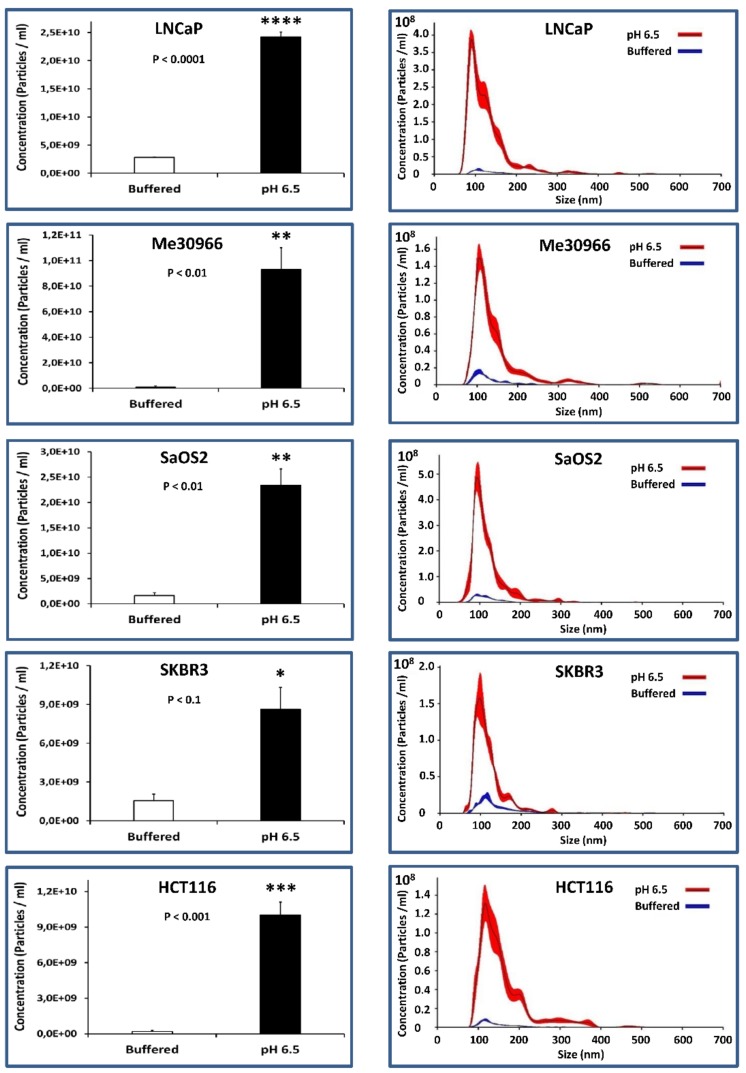
Nanoparticle tracking analysis (NTA) quantification of exosomes released from tumor cells cultured in buffered and pH 6.5 conditions. (**Left**) NTA analysis shows concentrations of particles isolated from cells in both culture conditions. Means ± SE of three different experiments are shown. The *p*-values were <0.1 in all cellular lines cultured in pH 6.5 with respect to buffered conditions. (**Right**) Overlay of the size, concentration, and distribution in both culture conditions. * *p* < 0.1, ** *p* < 0.01, *** *p* < 0.001.

To the purpose of quantifying the levels of exosomes in the various pH conditions, we cultured five human tumor cell lines of different histotypes for five days, and obtained the exosomes from the culture supernatants through serial ultracentrifugation; we then analyzed exosome purifications using the nanoparticle tracking analysis (NTA) technique (Nanosight NS300). Figure 4 (left panel) shows how the cells stabilized and cultured in an acidic medium release a greater number of exosomes as compared to the same cells in a physiological medium. The statistical analysis was performed using the unpaired *t*-test with the GraphPad Prism 6 software. Specifically, in HCT116 and Me30966 cells, the exosome release was 52- and 102-fold more, respectively, in acidic conditions as compared to 7.4 pH conditions, while it was 6-fold more in SKBR3 cells, 9-fold more in LNCaP cells, and 14-fold more in SaOS2 cells. The results are summarized in Table 2.

The profiles shown in Figure 4 (right panel) highlight that exosomes released by cells grown in acidic pH have a more homogeneous distribution and have a smaller size (121 ± 4 nm) as compared to exosomes released by the same cells grown in a buffered environment (147 ± 12 nm), which are more heterogeneous in size and distribution (Figure 4, right panel). In general, our results obtained with the NTA technique show that cells grown in acidic pH (6.5) produce an average of 22-fold more exosomes than the same cells grown in a buffered medium. This result was obtained by normalizing the number of exosomes obtained using NTA with the number of cells contained in the culture from which the exosomes were purified; more precisely, the value corresponds to the ratio between the averages of the numbers of exosomes produced by all cells cultured at pH 6.5 and the averages of the numbers of exosomes secreted by the same cells grown in buffered medium at pH 7.4.

### 2.4. Alkalization of the Tumor Cell Microenvironment Induces a Reduction of Exosome Release

**Figure 5:**
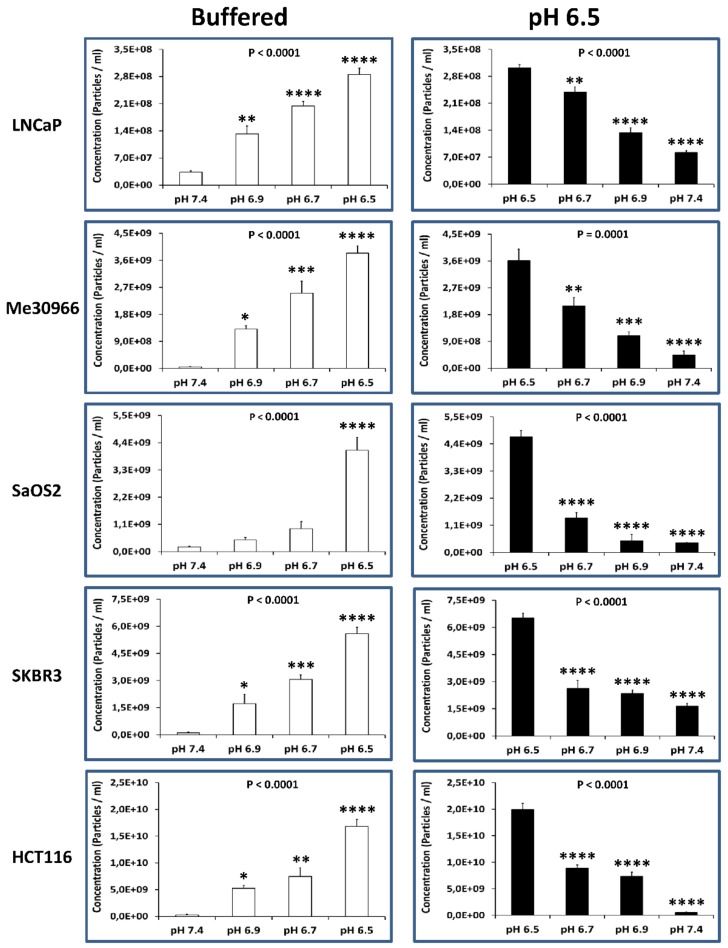
Alkalization of the tumor environment induces a reduction of exosome release. (**Left**) NTA analysis for the distribution and concentration of the number of exosomes released by the tumor cells cultured in pH conditions ranging from 7.4 to 6.5 (i.e., 7.4, 6.9, 6.7, and 6.5). Data are expressed as means ± SE of three independent experiments. The *p*-value was <0.0001 in all cellular lines cultured in acidic pH with respect to buffered conditions. (**Right**) NTA analysis for the distribution and concentration of the number of exosomes released by the tumor cells cultured in pH conditions ranging from 6.5 to 7.4 (i.e., 6.5, 6.7, 6.9, and 7.4). Data are expressed as means ± SE of three independent experiments. The *p*-value was ≤0.0001 in all cellular lines cultured in buffered conditions with respect to pH 6.5 conditions. * *p* < 0.1, ** *p* < 0.01, *** *p* < 0.001, **** *p*< 0.0001.

In this set of experiments, we evaluated the effect of different pH conditions on the exosome release by the various human tumor cell lines. To this purpose, we cultured the different cell lines at pH conditions ranging from 7.4 to 6.5 (i.e., 7.4, 6.9, 6.7, and 6.5). After five days of culture, the exosomes were purified from the cell-culture supernatant and then analyzed using NTA for their distribution and concentration. It is straightforward that as the pH of the microenvironment decreased (from 7.4 to 6.5; Figure 5, left panel) the number of exosomes released by the tumor cells increased 48–69-fold in SKBR3, Me30966, and HCT116cells, and 9–22-fold in LNCaP and SaOS2 cells.

To conversely demonstrate the pH dependence of exosome release, we started from acidic conditions (pH 6.5), progressively alkalinizing the culture medium in all the human tumor cell lines. Consistently with the experiments where we started from pH 7.4 conditions, we obtained here a progressive reduction of exosome release in all the cell lines (ranging from 4–40-fold lower when cultured at pH 7.4 as compared to the pH 6.5 conditions). The results obtained with NTA are shown in Figure 5 (right panel).

## 3. Discussion

A common feature of almost all tumors is the extracellular acidity due to lactic acid and H^+^ accumulation [21,22,23,24]. Previous reports suggested that the extracellular acidity of the tumor microenvironment increased the release of exosomes [6,28]. That was a key point in a better understanding of malignant tumors’ natural history, which goes through uncontrolled growth, local invasion, and metastasis, thus underlying the clinical progression. In fact, tumor-released exosomes were shown to contribute not only to the formation of the so-called metastatic or pre-metastatic niche, but also to the potential of inducing a tumor-like transformation in resident cells of metastatic organs, e.g., mesenchymal stem cells [12,34]. Thus, increased exosome release induced by extracellular acidity may represent an advantage exploited by malignant tumors to evade both the body reaction and any existing anti-tumor strategy. Between these advantages, we can list the ability of cancer cells to meet their energy needs via fermentation of sugars [14]; however, it seems highly conceivable that hypoxia and low nutrient concentrations transform cancer cells into fermenting anaerobes [13]. This hypothesis is also supported by the evidence that tumor cells under low nutrient supply or starvation feed on other cells (also called tumor cannibalism), in a way comparable to unicellular organisms [35]. This finding led to the discovery of a new oncogene, *TM9SF4*, which human malignant cells share with amoebae [36]. This suggests that tumor cells are more similar to unicellular microorganisms because, rather than cooperating with other cells, they are set to survive against all odds in an extremely hostile environment [13]. This survival equipment of cancer cells includes sugar fermentation in hypoxic conditions, proton pumps, and tumor cannibalism, as well as increased exosome release.

This study provides clear evidence, obtained using different techniques, that tumor microenvironment acidity induces an increase in exosome release in human tumor cells of different histotypes including prostate cancer, melanoma, osteosarcoma, colon cancer, and breast cancer. The use of cutting-edge techniques (NTA and nanoscale flow cytometry) for the identification, characterization, and quantification of extracellular vesicles allowed us to demonstrate that, in acidic pH, human tumor cells release a homogeneous population of nanosized extracellular vesicles (i.e., exosomes), while cells grown in a buffered environment release lesser levels of extracellular vesicles of a more heterogeneous size. Of course, the extracellular acidity has a crucial role in the cancer microevolutionism [37] and, here, we provide evidence that acidity induces morphological modifications in cancer cells, while they do not show an increase in their mortality. 

The mechanism underlying the increased release of nanovesicles in acidic conditions is not known. However, in our study, we exploited an entirely new experimental method for mimicking in vivo tumor conditions, that is, a progressive adaptation of the same tumor cell lines to acidic pH. This approach is consistent with the reproduction of the tumor microenvironment, and therefore, to the microevolutionary process that was recently hypothesized as a baseline pathogenetic mechanism of malignant tumors [38]. Through this mechanism, tumor cells that survive in very hostile microenvironments are the most suited to survive. The tumor acidity is probably the most important driving force of this adaptation. Malignant cancer cells are characterized by a primeval behavior, including cannibalism [39]. As far as exosome release is concerned, we know that one of the most important and recognized functions of exosomes is also to eliminate toxics [40], as we showed for chemotherapeutics [1], and it appears conceivable that low pH may trigger an increased exosome release to avoid the intracellular accumulation of toxics, and this is what we hypothesize to explain our results. 

Previous studies suggested that measurements of plasma levels of exosomes may represent a new and non-invasive diagnostic tool in the clinical follow-up of patients with tumors of different histologies [26,28]. The results of this study suggest that the selective pressure induced by the acidic microenvironment may lead to an abnormal release of exosomes expressing either unknown markers or known tumor markers, already shown to be expressed by human tumor-released exosomes, such as prostate-specific antigen (PSA) [28], carcinoembryonic antigen (CEA) [41], or melanoma antigen recognized by T cells 1 (MART-1) [42]. Therefore, this suggests that the presence of exosome-associated known tumor-associated molecules may represent a highly valuable diagnostic/prognostic tumor biomarker, together with exosome plasmatic levels, also called “circulating tumor mass” [31].

With the approach also described in this study, exosomes can be characterized and quantified in virtually all body fluids (e.g., blood, saliva, cerebrospinal fluid, breast milk, and urine) [3,4,26,27,43], thus providing an even more fruitful new clinical tool for follow-up of tumor patients.

All in all, we want to emphasize that, using different methods, while with different numbers, we demonstrated that acidic pH induces increased exosomes release by human cancer cells, independently of the nature of the primitive cancer. Using the most reliable method of quantifying and, at the same time, measuring the size of extracellular vesicles (i.e., NTA), we also showed that buffering the acidic tumor microenvironment induced a drastic reduction of exosome release. In a previous paper, we showed that the alkalinization of the tumor acidic microenvironment through the administration of a potent buffer (Basenpulver®; BP) led to a significant reduction of the growth of a very aggressive syngeneic melanoma in CB57/BL mice [19]. Furthermore, we demonstrated in a xenograft model that plasmatic levels of extracellular vesicles are related with the tumor size [26]. Lastly, in vivo treatment with proton-pump inhibitors reduced plasmatic exosome levels in comparable xenograft models [1]. These evidences strongly suggest that an alkalinizing approach should be included in future anti-cancer strategies, with the purpose to reduce exosome levels in cancer patients, with a potential to reduce the metastatic spread of tumors [12].

## 4. Materials and Methods 

### 4.1. Cell Lines

Metastatic melanoma Me30966 (obtained from Istituto Nazionale per lo Studio e la Cura dei Tumori, Milan, Italy), metastatic prostate carcinoma LNCaP, SaOS2 osteosarcoma, SKBR3 metastatic breast adenocarcinoma, and HCT116 colorectal carcinoma cell lines (all purchased from American Type Culture Collection (ATCC), Manassas, VA) were maintained in Rosewell Park Memorial Institute (RPMI-1640) medium supplemented with 10% fetal calf serum (FCS; Invitrogen, Milan, Italy) and antibiotics, at 37 °C in humidified 5% CO_2_. Experiments were performed in buffered medium at pH 7.4 and in RPMI-1640 medium without sodium bicarbonate (pH 6.9, 6.7, and 6.5) supplemented with antibiotics and 10% fetal calf serum (FCS; Invitrogen, Milan, Italy). Tumor cells were negative for mycoplasma contamination as routinely tested by PCR (Venor®GeM, Minerva Biolabs, Germany). The acidic cell-culture medium (pH 6.5) was obtained by the addition of 1 M HCl solution. The pH was measured with a pH 123 Microprocessor pH Meter (Hanna Instruments, Milan, Italy). All cell lines, before being grown in pH 6.5, were grown for a sufficient time in a non-buffered medium up to natural acidic conditions. Each cell line was slowly adjusted to pH 6.5 starting from unbuffered conditions (allowing tumor cells to acidify the microenvironment themselves) for five days, before measuring the pH at the end of the culture. At this point, the pH of the culture ranged from 6.5 (the majority of cell cultures) to 5 (melanoma cells). However, we established 6.5 for all tumor cell lines to standardize the experimental conditions and also to refer to in vivo measurements [16,44]. Thus, we progressively conditioned the pH of the cultures starting from 7.4, before passing to 7.2, 6.9, and finally to 6.5; this was obtained in a time ranging from three to four weeks, allowing the cells to not be exposed to short-term pH stress.

### 4.2. Exosome Purification from Cell-Culture Supernatants 

Supernatants from 1 × 10^6^ of each cell line were harvested after five days at 70–75% confluent cell cultures in 175-cm^2^ flasks, and exosomes were isolated as previously described [45]. After centrifugation of cells at 300× *g* for 5 min, supernatants were centrifuged at 1200× *g* for 15 min, followed by 12,000× *g* for 30 min. Supernatants were then centrifuged at 110,000× *g* for 1 h in a Sorvall WX Ultracentrifuge Series (ThermoFisher Scientific, Waltham, MA, USA) in order to pellet exosomes. After one wash in a large volume of phosphate-buffered saline (PBS), exosomes were resuspended in PBS (50 µL) for subsequent experimental analysis. In order to eliminate the exosomes from FCS, the FCS was filtered with 0.45-, and subsequently, 0.22-µm filters (Millipore Corp., Bedford, MA, USA), and then ultracentrifuged at 110,000× *g* before its addition to the culture media. For each technique (Bradford protein assay, NTA, nanoscale flow cytometry), the results were normalized to the number of the live cells at the end of the culture period, starting from the same cell number at the beginning of the culture and using the same volume of PBS for the analysis of the exosome preparation from each sample.

### 4.3. Nanoparticle Tracking Analysis

Nanoparticle tracking analysis (NTA) from Malvern (NanoSight NS300, Malvern Instruments, Malvern, UK) was used for size distribution and concentration measurements of nanovesicle samples in liquid suspension from the properties of both light scattering and Brownian motion. The NanoSight NS300 with a 405-nm laser instrument (Malvern Instruments, Malvern, UK) was used to detect nanovesicles. Five videos of typically 60-s duration were taken. Data were analyzed using the NTA 3.0 software (Malvern Instruments) which was optimized to first identify and then track each particle on a frame-by-frame basis. The Brownian motion of each particle was tracked using the Stokes–Einstein equation: D° = kT/6πηr, where D° is the diffusion coefficient, kT/6πηr = f_0_ is the frictional coefficient of the particle, for the special case of a spherical particle of radius r moving with uniform velocity in a continuous fluid of viscosity η, k is Boltzmann’s constant, and T is the absolute temperature. 

### 4.4. Flow Cytometry Analysis of Exosomes

Exosomes purified from cell-culture supernatants were diluted in PBS in a final volume of 40 µl (1 mg/mL). Anti-human CD81 allophycocyanin (APC) conjugated (Beckman Coulter; Brea, CA, USA) and mouse anti-human CD9 phycoerythrin (PE) conjugated (M-L13, RUO (GMP) BD Biosciences, USA) were added to the exosome preparation at optimal pre-determined concentrations and left for 30 min at room temperature (RT). The samples were then acquired on the CytoFLEX flow cytometer (Beckman Coulter, Brea, CA, USA). The cytometer was calibrated using a mixture of non-fluorescent silica beads and fluorescent (green) latex beads with sizes ranging from 110 nm to 1300 nm. This calibration step enabled the determination of the sensitivity and resolution of the flow cytometer (fluorescent latex beads) and the size of extracellular vesicles (silica beads). All samples were acquired at low flow rate for the same amount of time in order to obtain an estimate of absolute counts of exosomes comparable between various samples. The analysis of the data was performed with the FlowJo software (FlowJo, LLC; Ashland, OR, USA).

### 4.5. Western Blot

Briefly, subconfluent cells were lysed in CHAPS buffer 1× (Tris 10 mM pH 7.4, MgCl_2_ 1 mM, ethyleneglycoltetraacetic acid (EGTA) 1 mM, CHAPS 0.5%, glycerol 10%, and phenylmethylsulfonyl fluoride (PMSF) 1 mM) with protease (1 µg/mL leupeptin, 1 µg/mL pepstatin A, 1 µg/mL aprotinin, and PMSF 1 mM) incubated for 30 min on ice and centrifuged for 30 min at 12,000 rpm at 4 °C, thus removing cell debris and collecting the supernatant. Exosomes were lysed in CHAPS buffer 1× and processed as previously described. Protein concentration was determined using the Bradford protein assay (Bio-Rad Laboratories, Inc, Hercules, CA, USA). Thirty micrograms per sample were resolved on 10% acrylamide gel and transferred to a Protran BA85 nitrocellulose membrane (Schleicher & Schuell, Keene, NH, USA). Membranes were blocked overnight with 5% dry milk in PBS 1×. Blotting was performed employing anti-Alix (3A9, ThermoFisher Scientific, Waltham, MA, USA), anti-Tsg101 (4A10, GeneTex, Irvine, CA, USA), and anti-CD81 (B-11, Santa Cruz Biotechnology, Dallas, TX, USA) monoclonal antibodies. After incubation with appropriate peroxidase-conjugated anti-immunoglobulin G (IgG; Amersham Biosciences, Milan, Italy), membranes were revealed by enhanced chemiluminescence (Pierce, Rockford, IL, USA).

### 4.6. Confocal Analysis

The cells, after being fixed in 3% paraformaledehyde, were labeled with CD63 monoclonal antibody (MEM-259), and Alexa Fluor 488 (ThermoFisher Scientific) with a concentration of 1:25 for 2 h at room temperature, before being labeled with DAPI and observed using a confocal microscope. Images were taken by a FV1000 confocal microscope (Olympus, Tokyo, Japan), using a (Olympus) PlanApo objective 60× oil (numerical aperture 1.42). Excitation light was obtained using a DAPI laser (408 nm) for DAPI, and an argon-ion laser (488 nm) for Alexa 488; DAPI emission was recorded from 415 to 485 nm, and Alexa 488 emission was recorded from 495 to 550 nm. Images recorded had an optical thickness of 0.4 mm. 

### 4.7. Statistical Analysis

Results in the text are expressed as means ± standard error (SE), calculated using the GraphPad Prism software. The statistical analysis was done with an unpaired *t*-test (Student's *t*-test) and one-way ANOVA Bonferroni.

## 5. Conclusions

The ensemble of these reports with the data of this study suggest that (i) tumor acidity increases the release of extracellular vesicles by tumor cells, while also significantly lowering their size; (ii) either buffering, alkalinization, or anti-acidic treatment reduces the exosome release by tumors; (iii) exosome plasmatic levels are a measure of tumor progression with a potentially high clinical impact. 

## Figures and Tables

**Table 1:** Absolute number of CD9^+^/CD81^+^ exosomes in tumor cells cultured in buffered (pH 7.4) and pH 6.5 conditions by Nanoscale Flow Cytometry.

Nanoscale Flow Cytometry		
Tumor Cell Lines	Absolute Number of Exosomes (Mean ± Standard Error (SE))	
	Buffered (pH 7.4)	pH 6.5
**LNCaP**	4.9 × 10^3^ ± 2.6 × 10^3^	2.0 × 10^4^ ± 2.6 × 10^3^
**Me30966**	5.5 × 10^2^ ± 1.9 × 10^2^	3.0 × 10^3^ ± 3.5 × 10^2^
**SaOS2**	5.2 × 10^2^ ± 3.7 × 10	1.5 × 10^3^ ± 1.4 × 10^2^
**SKBR3**	2.7 × 10^3^ ± 1.5 × 10^2^	2.2 × 10^4^ ± 2.1 × 10^3^
**HCT116**	3.4 × 10^3^ ± 3.1 × 10^2^	8.3 × 10^3^ ± 3.5 × 10^2^

**Table 2:** Concentration of exosomes in tumor cells cultured in buffered (pH 7.4) and pH 6.5 conditions by Nanoparticle Tracking Analysis (NTA).

Nanoparticle Tracking Analysis (NTA)		
Tumor Cell Lines	Concentration of Exosomes (Particles/mL) (Mean ± SE)	
	Buffered (pH 7.4)	pH 6.5
**LNCaP**	2.8 × 10^9^ ± 3.9 × 10^7^	2.4 × 10^10^ ± 8.6 × 10^8^
**Me30966**	9.2 × 10^8^ ± 8.2 × 10^8^	9.3 × 10^10^ ± 1.7 × 10^10^
**SaOS2**	1.7 × 10^9^ ± 5.4 × 10^8^	2.3 × 10^10^ ± 3.2 × 10^9^
**SKBR3**	1.5 × 10^9^ ± 5.1 × 10^8^	8.6 × 10^9^ ± 1.7 × 10^9^
**HCT116**	1.9 × 10^8^ ± 1.1 × 10^8^	1.0 × 10^10^ ± 1.1 × 10^9^
